# Efficacy of the Nutritional Risk Index, Geriatric Nutritional Risk Index, BMI, and GLIM-Defined Malnutrition in Predicting Survival of Patients with Head and Neck Cancer Patients Qualified for Home Enteral Nutrition

**DOI:** 10.3390/nu14061268

**Published:** 2022-03-17

**Authors:** Zuzanna Przekop, Dorota Szostak-Węgierek, Magdalena Milewska, Mariusz Panczyk, Zuzanna Zaczek, Jacek Sobocki

**Affiliations:** 1 Department of Clinical Dietetics, Faculty of Health Sciences, Medical University of Warsaw, 01-445 Warsaw, Poland; dorota.szostak-wegierek@wum.edu.pl (D.S.-W.); magdalena.milewska@wum.edu.pl (M.M.); 2 Department of Education and Research in Health Sciences, Faculty of Health Sciences, Medical University of Warsaw, 00-581 Warsaw, Poland; mariusz.panczyk@wum.edu.pl; 3 Department of General Surgery and Clinical Nutrition, Centre of Postgraduate Medical Education, 00-401 Warsaw, Poland; zuzanna.zaczek@wum.edu.pl (Z.Z.); j.sobocki@mp.pl (J.S.); 4 Department of Human Nutrition, Faculty of Health Sciences, Medical University of Warsaw, 01-445 Warsaw, Poland

**Keywords:** survival analysis, head and neck cancer, home enteral nutrition, malnutrition, risk of malnutrition, NRI, GNRI, GLIM criteria

## Abstract

Malnutrition is a poor prognostic factor in cancer disease. In case of head and neck cancer, there are multiple disease symptoms and side effects of treatment that increase the risk of malnutrition. The aim of the study is to assess the association between nutritional status at the time of qualification for home enteral nutrition (HEN) and overall survival of patients with head and neck cancer (HNC), and assessment usefulness of selected nutritional indices as prognostic factors. The retrospective survival analysis involved 157 patients with HNC referred to HEN between January 2018 and October 2021. The nutritional status assessment was performed at the qualification for HEN visit. We have analyzed results of body mass index (BMI), Nutritional Risk Index (NRI) for patients <65 years, Geriatric Nutritional Risk Index (GNRI) for patients ≥65 years and malnutrition defined by Global Leadership Initiative on Malnutrition (GLIM). The mean patient survival was 44.7 weeks and the median was 23.9 weeks. Patients with low NRI and GNRI score had a higher risk of death (NRI: *p* = 0.0229; GNRI: *p* = 0.371). NRI, GNRI, and malnutrition defined by GLIM were superior to BMI as prognostic markers for survival. Results suggest that the use of NRI, GNRI, and GLIM criteria could provide useful prognostic information. The longer survival since the qualifying visit for home enteral nutrition suggests that nutritional management could be initiated earlier.

## 1. Introduction

Cancer-related malnutrition is still a common and unrecognized feature. It is estimated that 10 to 20% of cancer patients die due to malnutrition [1,2]. It is well-known that head and neck cancer (HNC) patients have multiple disease symptoms and chemotherapy, radiotherapy, or/and surgery side effects, such as dysphagia, dysgeusia, odynophagia, thick saliva or xerostomia increase the risk of malnutrition [3]. For that reason, nutritional status assessment and nutrition interventions play a crucial role in the management of patients with head and neck cancer. One of the methods used in HNC nutrition care is enteral nutrition, which is required mostly due to the cancer location, treatment side effects, and anorexia-cachexia syndrome [4,5].

According to European Society for Clinical Nutrition and Metabolism (ESPEN) home enteral nutrition (HEN) is recommended for high nutritional risk or malnourished patients and for those who are unable to meet nutritional requirements orally (energy intake, less than 60% requirement for 1–2 weeks) [6]. The Global Leadership Initiative on Malnutrition (GLIM) criteria were created to standardize the process of diagnosing malnutrition. As they are relatively new, there are few studies concerning the use of GLIM criteria, including survival analyses. Nutritional care during treatment helps to maintain or improve nutritional status, which has a positive impact on quality of life, reduces the toxicity of treatment, and lowers the risk of death [7]. The decision to start home enteral nutrition should also be made considering ethical aspects. In particular, in cancer patients, the predicted life expectancy may be short, and in such a case HEN would not prevent death from malnutrition, would be costly, useless, and could worsen the quality of life [8].

Poor nutritional status is an important prognostic factor in HNC and is related to the patient’s overall performance. There are a number of indices that are helpful in monitoring nutritional status. One of them is the Geriatric Nutritional Risk Index (GNRI) and the Nutritional Risk Index (NRI). They are simple and well-known nutritional tools, but they are usually analyzed as predictors of serious adverse events [9,10]. Yamahara et al. reported that the GNRI can be a good prognostic factor for survival in head and neck cancer, but the authors used this index regardless of patient age [11]. To the best of our knowledge, there is no work examining GLIM criteria, NRI, and GNRI as prognostic factors for survival of patients with head and neck cancer on home enteral nutrition.

In this study we investigated the impact of nutritional status at the time of qualification for HEN on overall survival in patients with head and neck cancer and the usefulness of selected nutritional indices (BMI, NRI, GNRI, and GLIM criteria) as prognostic factors.

## 2. Materials and Methods

### 2.1. Study Design and Population

We recruited patients qualified for home enteral nutrition (HEN) in the national referral center between January 2018 and October 2021. Inclusion criteria were age 18 years or older and diagnosed head and neck cancer. All patients were referred to HEN by general practitioners or another physician and signed an informed consent for enrollment at HEN procedure. Data from qualification visit and survival information were collected prospectively, and all medical records were retrospectively reviewed. Patients whose medical records were missing some data required for this study, patients who were still in HEN procedure but whose observation period was less than 6 months, patients who were lost to follow-up, and those who were transferred to hospice were excluded. Patients with tonsil cancer were also excluded, based on a performed simulation that showed that they disturbed the group picture. Finally, a group of 157 patients was evaluated. The study was approved by the Ethics Committee of the Medical University of Warsaw (KB/87/2018).

### 2.2. Institutional Approach to Home Enteral Nutrition Procedure

Routine institutional practice to qualify for home enteral nutrition included a nutritional assessment, patient or caregiver education on tube feeding administration methods, and enteral nutrition access care blood tests, physical examination, and prescription of an enteral nutrition formula in the amount appropriate to the patient’s nutrient and fluid needs.

Nutritional assessment included height and body weight measurements and was performed by a trained dietitian or nurse. Body weight was measured according to the recommendations of CDC, using a portable electronic scale (Fawag S.S. model ZOL-3.4. w. WTL, Lublin, Poland) with an accuracy of 0.1 kg [12].

### 2.3. Data Collection and Nutritional Status Evaluation

The collected data included sex, age at qualification to home enteral nutrition, tumor site, weight, height, BMI, unintentional weight loss in 6 months at qualification visit, serum albumin level, and C-reactive protein (CRP). For the time-to-event analysis, the date of the qualifying visit and the date of the patient’s death were determined.

BMI categories for patients < 65 years were classified according to the CDC criteria: underweight (BMI < 18.5 kg/m^2^), normal weight (BMI ≥ 18.5 kg/m^2^–< 25.0 kg/m^2^), overweight (BMI ≥ 25.0 kg/m^2^) [12], and Lipschitz criteria for patients ≥ 65 years: underweight (BMI < 22.0 kg/m^2^), normal weight (BMI ≥ 22.0 kg/m^2^–< 27.0 kg/m^2^), overweight (BMI ≥ 27.0 kg/m^2^) [13]. The percentage of weight loss in 6 months was calculated according to the following formula:

(1) $$\%\;\mathrm{weight}\;\mathrm{loss}=\frac{\mathrm{weight}\;\mathrm{change}\;\left(\mathrm{kg}\right)}{\mathrm{current}\;\mathrm{weight}\;\left(\mathrm{kg}\right)+\mathrm{weight}\;\mathrm{change}\;\left(\mathrm{kg}\right)}\times 100$$

Based on the collected data, the GLIM criteria were used to assess the nutritional status of patients [14]. As the data on muscle mass loss were not available, only weight loss and BMI were assessed in the phenotypic GLIM criteria. Low body mass index was defined as follows: <20 kg/m^2^ if <70 years or <22 kg/m^2^ if ≥70 years and weight loss > 5% within past 6 months. The etiological criteria of GLIM are reduced food intake and inflammation. In case of inflammation, it was defined as CRP > 10 mg/L. The criterion of reduced food intake was considered as met for all cases included in the analysis, as all patients were at the time of admission on home enteral nutrition. The severity of malnutrition was then assessed. Moderate malnutrition was diagnosed if any of the following criteria were met: weight loss < 5% within the last 6 months or BMI < 20 kg/m^2^ at age < 70 years or <22 kg/m^2^ at age ≥ 70 years. Severe malnutrition was diagnosed if there was weight loss > 5% within the last 6 months or BMI < 18.5 kg/m^2^ at age < 70 years or <20 kg/m^2^ at age ≥ 70 years.

Based on serum albumin level, body weight, and ideal body weight value, Nutritional Risk Index (NRI) was calculated in patients younger than 65 years and Geriatric Nutritional Risk Index (GNRI) in patients ≥65 years. Ideal body weight was calculated based on Lorentz formula:

Ideal body weight (men) = height − 100 − ((height − 150)/4)

Ideal body weight (women) = height − 100 − [(height − 150)/2](2)

The patients with NRI score of >100 were considered to be at no nutritional risk, 97.5–100 as at mild risk, 83.5–97.5 at moderate, and <83.5 at major nutritional risk. NRI was calculated as follows:

NRI = (1.519 × serum albumin ) (g/L) + 41.7 × (present weight/ideal bodyweight)(3)

Patients over 65 years old were divided into the four grades of nutrition related-risk (GNRI > 98—no risk, 92 to ≤98—low risk, 82 to <92—moderate risk and <82—major risk). The GNRI was calculated based on the formula proposed by Bouillanne et al. [15]:

GNRI = (1.489 × serum albumin ) (g/L) + 41.7 × (present weight/ideal bodyweight)(4)

### 2.4. Statistical Analysis

The collected data were presented using the descriptive statistics. The variables quantitative were described using the mean and standard deviation and categorical variables using the number (*n*) and frequency (%).

Mean values of selected parameters were compared using the Student’s *t*-test for independent groups. Whereas the contingency tables along with the Pearson’s chi-squared test were used to test null hypothesis about no difference in distribution of a categorical variable between two independent groups.

Survival analysis was performed using Kaplan–Meier method. The log rank test was used to test the null hypothesis of no difference in survival between more independent groups. On the other hand, multivariate Cox regression analysis was used to assess the influence of selected factors on overall survival. A hazard ratio (HR) was estimated for each factor along with a 95% confidence interval.

All calculations were performed using STATISTICA version 13.3. (TIBCO Software Inc., Palo Alto, CA, USA). The null hypothesis was tested with a priori set significance level of 0.05.

## 3. Results

A total of 157 patients met the inclusion criteria of the study. The mean age of the group was 63.8 ± 11.12 years and 52.9% of the patients were under 65 years of age. Women constituted 25.5% of the study group. All patients had head and neck cancer and the cancer types were distributed as follows: 40.1% (*n* = 63) oral cavity, 18.5% (*n* = 29) oropharynx, 17.8% (*n* = 28) larynx, 14.0% (*n* = 22) hypopharynx, 4.5% (*n* = 7) nasopharynx, 2.6% (*n* = 4) nasal cavity and paranasal sinuses, 1.3% (*n* = 2) salivary glands, and 1.3% (*n* = 2) other types. There were no significant differences in tumor location between sex and age groups. During the visit, the enteral diet formula was established. Twenty-two different diets were used. A normocaloric formula was prescribed for 82.8% of the patients. Almost half of them (42.6%) were enriched with fiber, which constituted 35.0% of the total group, and 15.9% of the patients received a hypercaloric diet. The average caloric content of the diet was 1449 ± 257 kcal daily. One hundred thirty-nine patients (88.5%) had percutaneous endoscopic gastrostomy (PEG). The mean body weight of group was 59.7 ± 13.23 kg, and the mean body weight loss in last 6 months was 11.8 ± 7.44 kg. Group characteristics, divided by sex and age are shown in Table 1.

The results of risk of malnutrition assessed with NRI (patients below 65 years) and GNRI (patients ≥65 years old) are shown in Table 2. In both these groups, there was no significant difference between categories of malnutrition risk and sex (*p* = 0.667 for NRI and *p* = 0.531 for GNRI).

**Figure 1:**
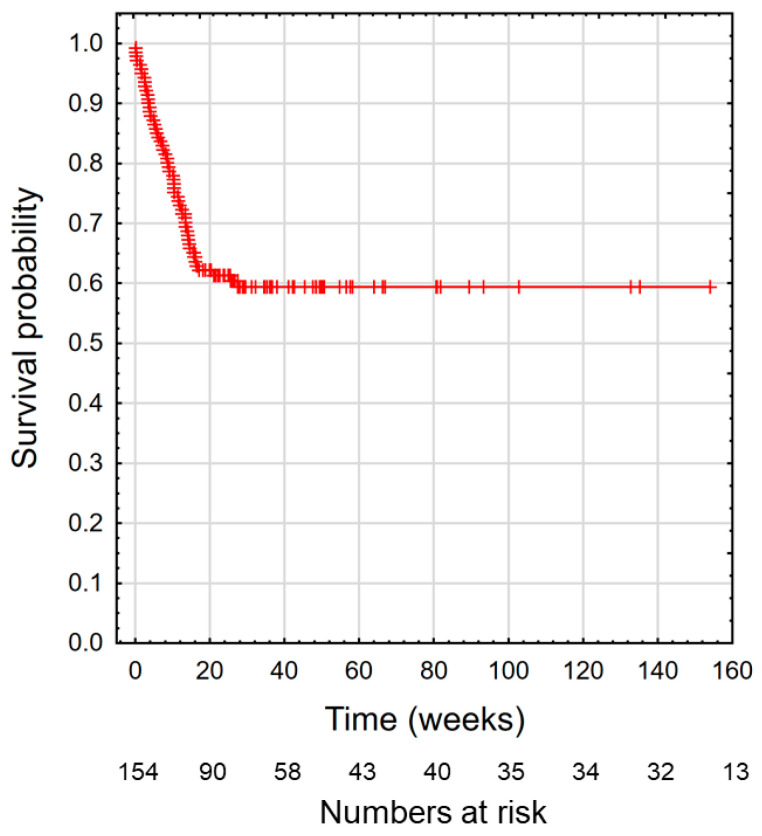
Cumulative curve.

**Figure 2:**
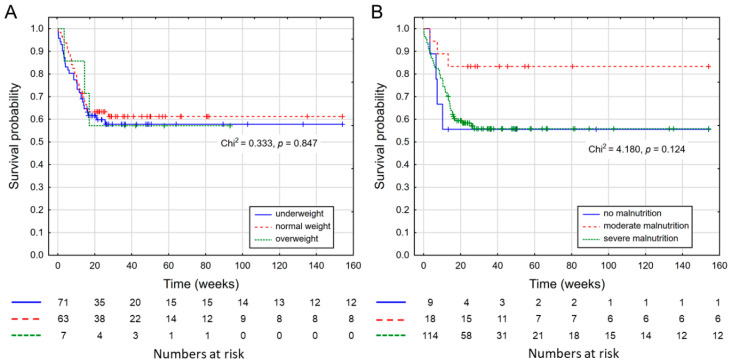
Survival curves of all subjects; (**A**) according to BMI; (**B**) according to GLIM.

The overall survival curve for 157 patients who were qualified for home enteral nutrition is shown in Figure 1. The mean patient survival was 44.7 weeks and the median was 23.9 weeks. Figure 2 shows the survival curve depending on BMI and GLIM classification at qualification visit. The survival time in all figures is presented in weeks.

**Figure 3:**
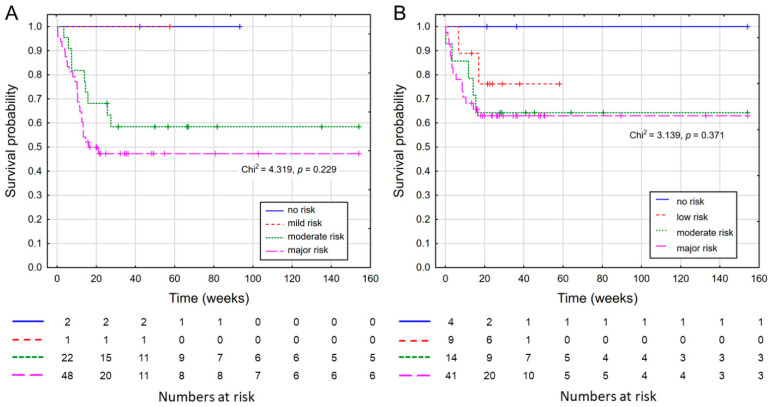
Survival curves (**A**) according to NRI; (**B**) according to GNRI.

As NRI and GNRI are age-dependent indicators of malnutrition risk, the analyzed group was as follows: NRI, patients below 65 years old (*n* = 83); GNRI, patients ≥65 years old (*n* = 74). The results for NRI and GNRI are shown in Figure 3. 

The results of multivariate Cox regression analysis showed that CRP level was positively related to the risk of death (*p* < 0.001; HR = 1.015; 95% CI, 1.01–1.02). In case of BMI the results were as follows: *p* = 0.727 (95% CI, 0.91–1.14), for percentage of body weight loss: *p* = 0.532 (95% CI, 0.83–1.10), and for body weight loss in kilograms: *p* = 0.480 (95% CI, 0.78–1.11). The results of analysis for GLIM-defined no malnutrition vs moderate malnutrition showed the relationship but the strength of the result was weak (*p* = 0.082, HR = 0.260, 95% CI, 0.06–1.20). There was no significant difference between no malnutrition and severe malnutrition (*p* = 0.683, HR = 0.629, 95% CI, 0.18–2.22).

## 4. Discussion

The European Society for Clinical Nutrition and Metabolism (ESPEN) recommends regular screening of malnutrition risk to detect disturbances at an early stage for all cancer patients [1]. Enteral nutrition is used for head and neck cancer patients due to the frequent inability to meet nutritional needs orally. Gazcon-Ruiz et al. pointed out that the GLIM criteria for malnutrition in patients with head and neck tumors have a higher sensitivity than the ESPEN criteria and may be helpful in the early detection of malnutrition [16]. In our study, based on the GLIM definition of malnutrition, 94.2% of the group was malnourished. The results show that 76.4% of all patients were severely malnourished and 17.8% had moderate malnutrition during the home enteral nutrition qualification visit. It suggests that an earlier referral for nutritional treatment could be beneficial for patients.

Paccagnella et al. reported that the median survival of patients with head and neck cancer who were on home enteral nutrition was 5.4 months and the duration of HEN was 118 days, and only 4.5% of HNC patients resumed oral nutrition [17]. In our study, the mean patient survival was 44.7 weeks and the median was 23.9 weeks.

To the best of our knowledge, this is the first study analyzing GLIM defined malnutrition, NRI, and GNRI as a prognostic value in a group of patients with head and neck cancer receiving home enteral nutrition. In our study, there were no statistically significant differences between the categories of studied tools. However, we did not have information on the time of diagnosis of head and neck cancer and its stage. Our results suggest that BMI value, which is commonly used in clinical practice, is a weak tool for predicting survival of analyzed group of patients. Similar results, suggesting that BMI cannot be considered as a predictor without an assessment of body composition were reported by Nazari et al. [18]. GLIM-defined malnutrition, NRI and GNRI indices are better in assessing patient prognosis. Prediction of survival is of critical importance. The ESPEN guidelines recommend that the issues that should be considered before initiating home enteral nutrition are quality of life and estimated survival [1]. Yin et al. reported that malnutrition defined by GLIM has prognostic significance in predicting survival in cancer patients with newly diagnosed cancer (*p* < 0.001) [19]. A similar result was reported in patients with gastric cancer (*p* = 0.0084) [20]. In our study, the difference is nonsignificant (*p* = 0.124) and the group without malnutrition seems to bias the results. This may be due to the fact that only 9 out of 157 patients were in the no malnutrition category and information on muscle mass loss was not available. There are fewer data on the relationship between NRI/ GNRI values and survival in head and neck cancer patients. In the case of GNRI, there are only two studies that have examined its association with mortality in this patient population [9,11]. However, Nakayama et al. used the GNRI regardless of patient age [9]. In these two studies, the authors found that a high risk of malnutrition assessed with GNRI was associated with a worse prognosis. Our results seem to confirm this, but the strength of the results is weak (*p* = 0.371). Previous studies have examined the benefit of NRI use in patients receiving total parenteral nutrition, in patients with heart failure, and in patients with renal disease [21,22,23,24]. There are fewer studies that have comprehensively evaluated the performance of NRI in cancer, especially in head and neck cancer. Our results indicate that NRI is more sensitive than BMI to assess mortality risk in patients with head and neck cancer who receive HEN. Bao et al. reported that BMI, albumin level, prognostic nutritional index, and NRI had prognostic value in patients with oral cavity cancer. Similarly to our results, BMI had the smallest predictive value [25].

Our study had some limitations. First, because of the retrospective design of the study, we did not have access to information on cancer stage, time of cancer diagnosis and treatment, as these are not routinely collected. Second, we did not have information on the muscle mass loss, which could affect the results concerning malnutrition defined by GLIM, especially in patients who were classified in the no malnutrition group. Furthermore, we had no information on the exact cause of death. Moreover, in our study, we demonstrated only association and not causality. However, control randomize trial investigating nutritional intervention in this group is inconsistent with the principles of ethics. A strength of our analysis is that our study was conducted in a national reference center of HEN and is one of the first studies to analyze GLIM-defined malnutrition, NRI, GNRI, as prognostic markers, and the first in a group of patients with head and neck cancer receiving home enteral nutrition.

## 5. Conclusions

The study has shown that patients with low NRI and GNRI score had a higher risk of death. Moreover, NRI, GNRI, and malnutrition defined by GLIM were superior to BMI as prognostic markers for survival. These results suggest that the use of NRI, GNRI, and GLIM criteria could provide useful prognostic information. The survival duration since the qualifying visit for home enteral nutrition suggests that nutritional management should be initiated earlier.

## Figures and Tables

**Table 1:** Characteristics of the study group divided by sex and age.

Variable	Total Group		Female (*n* = 40)				*p*-Value *	Male (*n* = 117)				*p*-Value *	*p* Difference between Genders *
			<65 (*n* = 21)		≥65 (*n* = 19)			<65 (*n* = 62)		≥65 (*n* = 55)			
	M	SD	M	SD	M	SD		M	SD	M	SD		
body weight (kg)	59.7	13.23	52.5	11.31	50.3	10.90	0.529	61.3	11.23	64.0	14.25	0.260	0.000
% loss of body weight	16.3	9.11	15.7	10.56	14.6	8.32	0.718	16.9	7.99	16.3	10.08	0.733	0.408
BMI (kg/m^2^)	20.6	3.88	19.7	3.89	20.3	3.47	0.629	20.2	3.60	21.4	4.23	0.084	0.270
**BMI Categories**	***n***	**%**	***n***	**%**	***n***	**%**	***p*** -Value **	***n***	**%**	***n***	**%**	***p*** -Value *	*p* Difference between Genders **
Normal weight	70	46.5	10	47.6	7	36.8	0.120	35	56.5	18	32.7	0.024	0.854
Underweight	73	44.6	8	38.1	12	63.2		21	33.9	32	58.2		
Overweight	14	8.9	3	14.3	0	0.0		6	9.7	5	9.1		
GLIM													
No	9	5.7	2	9.5	1	5.3	0.841	3	4.8	3	5.5	0.564	0.538
Moderate	28	17.8	5	23.8	4	21.1		8	12.9	11	20.0		
Severe	120	76.4	14	66.7	14	73.7		51	82.3	41	74.6		
CRP													
<10 mg/L	28	17.8	8	38.1	2	10.5	0.044	12	19.4	6	10.9	0.206	0.170
≥10 mg/L	129	82.2	13	61.9	17	89.5		50	80.7	49	89.1		

M-mean, SD—standard deviation, BMI—body mass index, CRP—C-reactive protein, * Student’s *t*-test, ** Pearson’s chi-squared test.

**Table 2:** Summarize of NRI and GNRI results.

Variable	NRI (*n =* 83)		GNRI (*n* = 74)	
	*n*	%	*n*	%
No risk	2	2.4	5	6.8
Mild/Low risk	2	2.4	10	13.5
Moderate risk	28	33.7	17	23.0
Major risk	51	61.5	42	56.8
